# On-Surface Synthesis of Na-Porphyrins Using NaCl as a Convenient Na Source

**DOI:** 10.1021/prechem.3c00014

**Published:** 2023-04-05

**Authors:** Zewei Yi, Chi Zhang, Zhaoyu Zhang, Rujia Hou, Yuan Guo, Wei Xu

**Affiliations:** Interdisciplinary Materials Research Center, School of Materials Science and Engineering, Tongji University, Shanghai 201804, P. R. China

**Keywords:** metallo-porphyrin, alkali metal, on-surface chemistry, scanning tunneling microscopy, density functional theory

## Abstract

Metallo-porphyrins with different metal centers display unique properties and are essential in various biological and chemical processes. Enormous efforts have been devoted to enriching the family of metallo-porphyrins on surfaces mainly through metalation processes within porphyrins and exogenous pure metals or intrinsic surface adatoms, which have focused on transition elements. However, less attention has been paid to the synthesis of alkali-metal-based porphyrins on a surface. Herein, by a combination of scanning tunneling microscopy (STM) imaging/manipulations and density functional theory (DFT) calculations, we report the fabrication of Na-porphyrins on Au(111) by introducing NaCl, i.e., two double-layered Na-centered porphyrins. Moreover, the interconversion between them was realized by precise STM manipulations. Our results demonstrate the feasibility of metalation by applying inorganic salt, which would serve as a promising strategy to embed intramolecular metal components into porphyrins for further functionalization and modification.

## Graphical abstract

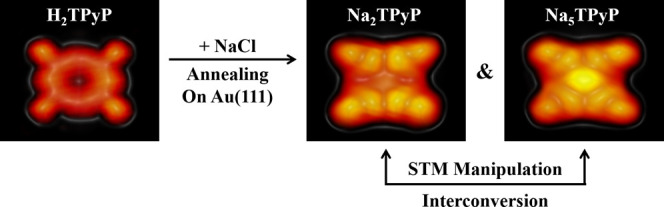

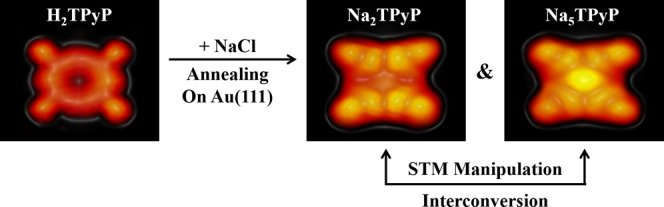

Metallo-porphyrins (M-porphyrins), a type of porphyrin with a metal center embedded in the macrocycle, participate in numerous biological processes in nature,^1^ typically as chlorophyll in photosynthesis and heme in small-molecule storage and transport. Owing to the metal center involved, M-porphyrins also turn out to be valuable in many physical and chemical processes, for example, acting as nanomagnets^2,3^ and catalysts.^4,5^ In the last few decades, tremendous efforts have been devoted to the synthesis of M-porphyrins with various central atoms, which cover most of the metal elements in the periodic table,^6^ to achieve tailored properties. In the field of surface science, the metalation processes of porphyrins on surfaces^7^ have also been extensively explored with the aid of advanced surface techniques^8,9^ to provide submolecular insights. Previous studies have realized the synthesis of M-porphyrins with transition metals (e.g., Fe,^10^ Co,^11^ Cu,^12^ and Ru^13^), lanthanides (e.g., Ce^14^), and actinides (e.g., Th^15^) on surfaces. Recently, the family of M-porphyrins was further expanded with the successful introduction of main-group elements, including Ge,^16^ alkaline-earth metal Mg,^17^ and nonmetal element Si.^16^ However, on-surface synthesis of alkali-metal-based M-porphyrins has been less reported.^18^ Alkali metals have been proved to modify the physical properties of molecular complexes^19,20^ as good dopants, as well as to promote various essential catalytic processes.^21,22^ Therefore, it is of general interest to enrich the family of M-porphyrins with alkali metal elements, which would pave the way for further modification of porphyrins in various fields including but not limited to biology and catalysis.

To synthesize M-porphyrins from the initial porphyrin (2H-porphyrin) state on surfaces, the most commonly used strategy is the deposition of target metal atoms, which are further incorporated as metal centers,^11,14–16^ while the pure metal sources are usually sensitive to air or have high sublimation temperatures. Another approach is the direct capture of surface adatoms,^10,12,17^ which is limited by the kinds of substrates. Interestingly, sublimation of metal–organic complexes, such as (Ru_3_(CO)_12_),^13^ has shown its availability of providing metal sources as an alternative method. It is thus highly desirable to introduce some easily available metal sources to achieve porphyrin metalation.

In this study, sodium chloride (NaCl), which generally serves as an insulating support^23,24^ on surfaces and a feasible source to introduce the alkali metal Na to interact with organic molecules^25,26^ via ionic interactions, was applied to induce the metalation of porphyrins (forming Na-porphyrins) on Au(111). The tetrapyridyl-porphyrin molecule (shortened as H_2_TPyP; see Scheme 1) was selected as the porphyrin precursor. By a combination of high-resolution STM imaging/manipulations and DFT calculations, we showed the synthesis of two double-layered Na-centered porphyrins, namely, Na_5_TPyP (in majority) and Na_2_TPyP (cf. Scheme 1), on Au(111) using NaCl. The control experiment by introducing pure alkali metal Na also gave access to such a metalation, with the fabrication of the same Na-porphyrins, which verified the versatility of NaCl in inducing porphyrin metalation. Moreover, by applying STM lateral manipulations, the interconversion between Na_5_TPyP and Na_2_TPyP was achieved. These findings demonstrated a feasible on-surface synthesis protocol to form M-porphyrins with alkali metal incorporated, which supplements fundamental understandings of alkali-metal-based porphyrins and would serve as a promising strategy to introduce intramolecular metal components.

**Scheme 1:**
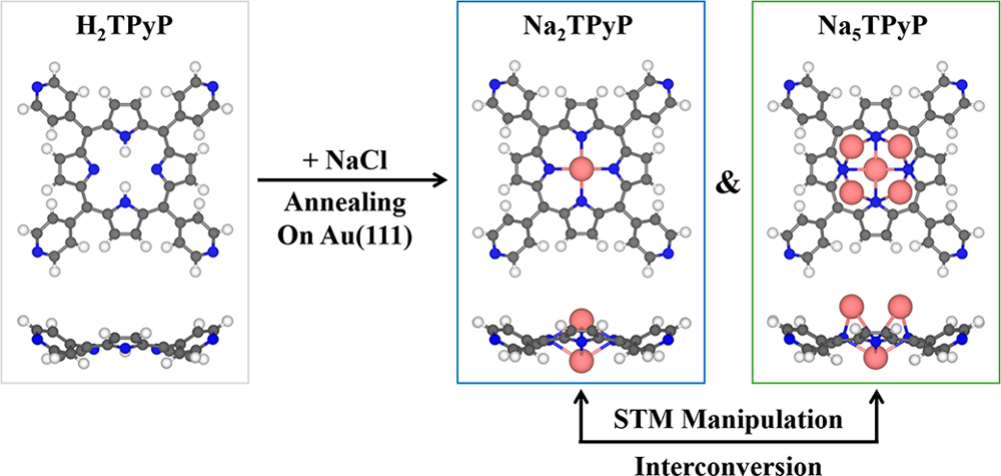
Schematic Illustration Showing the Fabrication of Na-Porphyrins on Au(111) by Introducing NaCl (H: White; C: Gray; N: Blue; Na: Pink)

Deposition of H_2_TPyP molecules onto Au(111) followed by annealing at ∼510 K led to the formation of a porous structure (Figure 1a). The close-up STM image (Figure 1d) clearly showed the arrangement of molecules involved, where four neighboring ones were aligned in a rhomboid pattern and two adjacent ones were perpendicular to each other (with individual molecules indicated by white rectangles). The morphology of each molecule remained unchanged compared to that obtained after deposition at ∼300 K, as was featured by an empty core and four bright ends (the tilted pyridyl groups).^3,27^ In addition, the rectangular molecular morphology originates from the well-known saddle-shaped adsorption geometry of the *meso*-substituted porphyrins on surfaces to avoid the intramolecular steric hindrance (Figure S1).^27^ Notably, the self-metalation of porphyrins was reported to take place at a higher temperature after cyclodehydrogenation on Au(111).^28^ Thus, the porphyrin molecules were still intact at ∼510 K in our study. The corresponding structural model is shown in Figure 1g.

**Figure 1:**
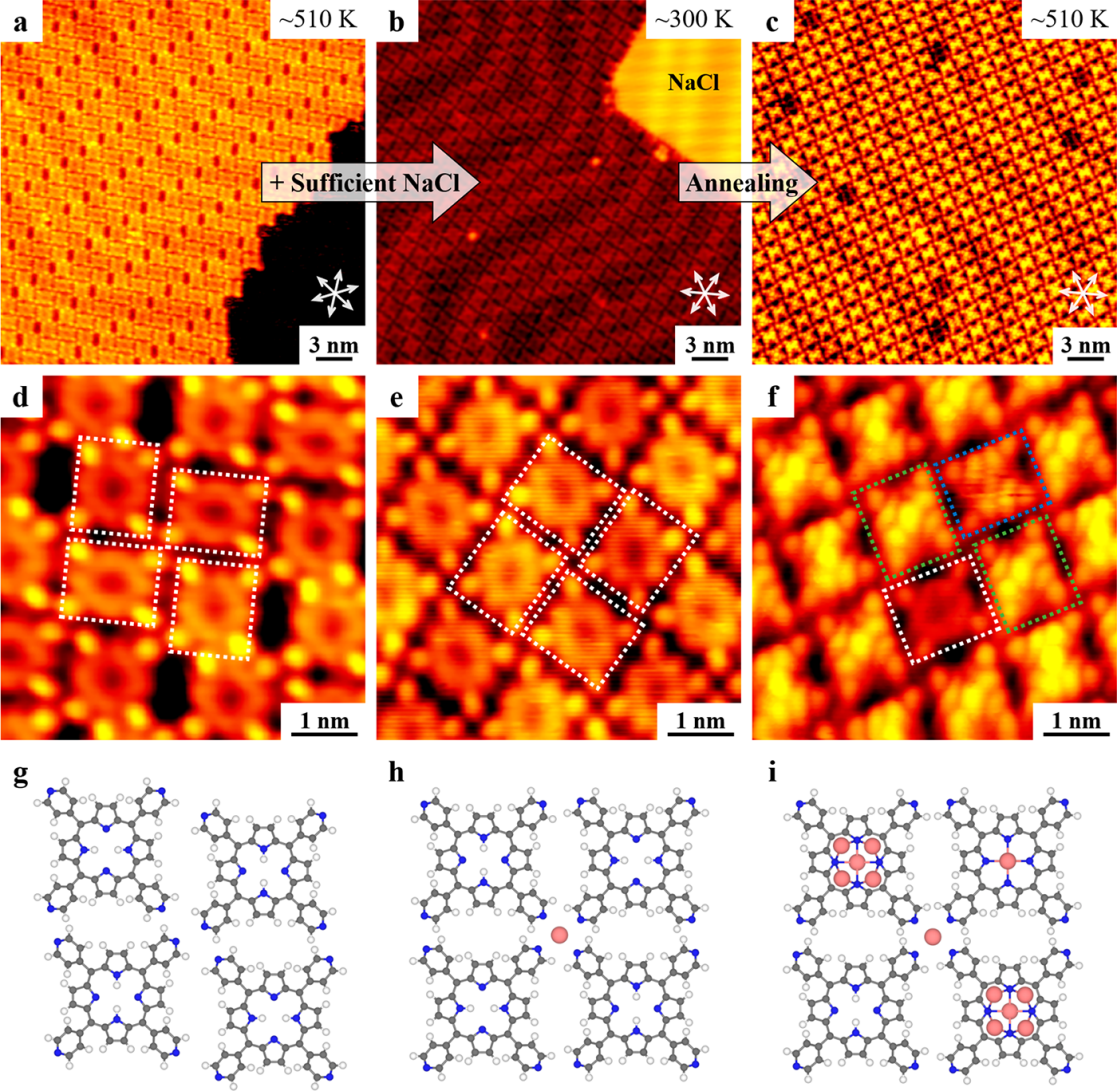
Aggregation and metalation processes of H_2_TPyP molecules on Au(111). (a) Large-scale and (d) close-up STM images showing the porous self-assembled structure after the deposition of H_2_TPyP and annealing at ∼510 K. (b) Large-scale STM image showing the coexistence of close-packed molecular islands and NaCl islands after the deposition of NaCl at ∼300 K. (e) High-resolution STM image of the close-packed structure. (c) Large-scale and (f) close-up STM images showing the formation of metalated molecules after annealing at ∼510 K. The individual H_2_TPyP and two types of metalated molecules (with dim or bright features at the center) are indicated by white, blue, and green rectangles, respectively. The close-packed directions of the Au(111) substrate are indicated by white arrows. Scanning conditions: *V*_*t*_ = −1.2 to −1.5 V, *I*_*t*_ = 0.6 nA. (g–i) Structural models of the depicted four neighboring molecules in (d–f). H: white; C: gray; N: blue; Na: pink.

Next, to examine the feasibility of applying NaCl to synthesize Na-porphyrins, sufficient NaCl was introduced to the sample at ∼300 K, which resulted in the coexistence of molecular structures and NaCl islands (Figure 1b). It is noteworthy that after introducing NaCl, the porous H_2_TPyP structure (Figure 1a and d) transformed to the close-packed one (Figure 1b and e), and every four adjacent molecules were aligned in a square pattern (indicated by white rectangles). Close inspection of the individual molecules involved (Figure 1e) showed that the morphology was consistent with the characteristics of H_2_TPyP as discussed above. Moreover, a dim protrusion was visible at the center of every four neighboring H_2_TPyP molecules in a special tip state (Figure S2a) and was attributed to Na^26^ according to the previous work.^29^ Na atoms interacted with the four surrounding N atoms of pyridyl rings and gathered four H_2_TPyP molecules together, resulting in the close-packed arrangement (Figure 1h). Such a structural transformation process displays the aggregation effect provided by NaCl, where Na is capable of interacting with negatively charged atoms (such as N^29^ and O^26^) from specific organic molecules by forming intermolecular ionic interactions.

Thereafter, the above sample was annealed at ∼510 K, and interestingly, a majority of the molecules turned bright at the center, as shown in Figure 1c. From the close-up STM image (Figure 1f), three different types of porphyrin-based molecules could be distinguished, while the molecular arrangement remained the same (Figures 1i and S2b). Apart from the intact one (in the white rectangle) with a characteristic empty core, two newly formed species with dim (in the minority) and bright (in the majority) features at the center of the macrocycles appeared and were temporarily named dim and bright molecules (as depicted by blue and green rectangles, respectively). It is well-known that H_2_TPyP molecules can serve as a good host,^30^ while a Au(111) substrate is able to provide Au adatoms as metal centers for metalation of porphyrins.^28^ Nevertheless, no self-metalation of H_2_TPyP molecules was observed on Au(111) at the same annealing temperature of ∼510 K (see Figure 1a and d). Thus, the possibility of formation of AuTPyP under this condition was ruled out. Moreover, considering that the dim and bright molecules appeared only after deposition of NaCl, we concluded that NaCl must play an important role in the formation of both species.

To explore the structures of both porphyrin-based species, DFT calculations were performed. The energetically favorable structural models on Au(111) together with the corresponding STM simulations are shown in Figure 2, in comparison with the high-resolution STM images of the three species. As shown in Figure 2d, the H_2_TPyP molecule adsorbed on Au(111) adopts a saddle-shape configuration as reported,^3,27^ resulting in the appearance of four bright protrusions at the edges of the molecule in morphology (Figure 2a). With the absence of a metal center, the initial H_2_TPyP molecule appeared with an empty black center, which was reproduced by the STM simulation (Figure 2g). As for the structures of Na-porphyrins, the possibilities with an increasing number of Na atoms embedded in the macrocycle were systematically considered (Figure S3). When one Na atom was incorporated into the macrocycle, forming NaTPyP (Figure S3a), a neglectable black dot appeared at the center in the simulated STM image (Figure S3f), which did not match with the obvious features of either species. Further involvement of more Na atoms led to the adsorption on the top layer of the NaTPyP structure. When two Na atoms were involved (see Figure 2e), the additional Na was located at the center of the top layer, forming Na_2_TPyP, and a dim protrusion appeared at the molecular center in the simulated STM image (Figure 2h), in line with the feature of a dim molecule (Figure 2b). Such a bipyramidal configuration agrees well with the structures of alkali-metal-based porphyrins reported in solution chemistry^31^ and on surfaces.^18^ Thus, the dim molecule was attributed to Na_2_TPyP. As for the case of Na_3_TPyP (Figure S3c), although the molecular center became brighter, the two additional Na atoms interacted with N from the macrocycle, leading to an obvious symmetry along the diagonal line (Figure S3h), which was out of line with the experimental observation. When the number of additional Na atoms increased to three forming Na_4_TPyP, an obvious asymmetry could be expected, and thus the possibility was ruled out. Only when Na_5_TPyP was formed could the characteristic morphology of the bright molecule (Figure 2c) be nicely reproduced by the corresponding STM simulation (Figure 2i). Further increasing the number of Na atoms to form Na_6_TPyP resulted in the huge bright feature at the center, which almost covered up the whole molecule and was thus ruled out. Moreover, the situation with the adsorption of Cl on the top of Na-porphyrin was also excluded, which will be further discussed in the following text. In addition, the DFT-simulated STM images obtained at the typical bias voltages agree well with the corresponding characteristic STM topographies (Figure S4), further verifying the assignment of the molecular species. Accordingly, the bright molecule was attributed to Na_5_TPyP, where each Na at the top layer evenly bonded to two N atoms of the porphyrin core (Figure 2f), leading to the much higher apparent height and appearance of a uniform bright protrusion.

**Figure 2:**
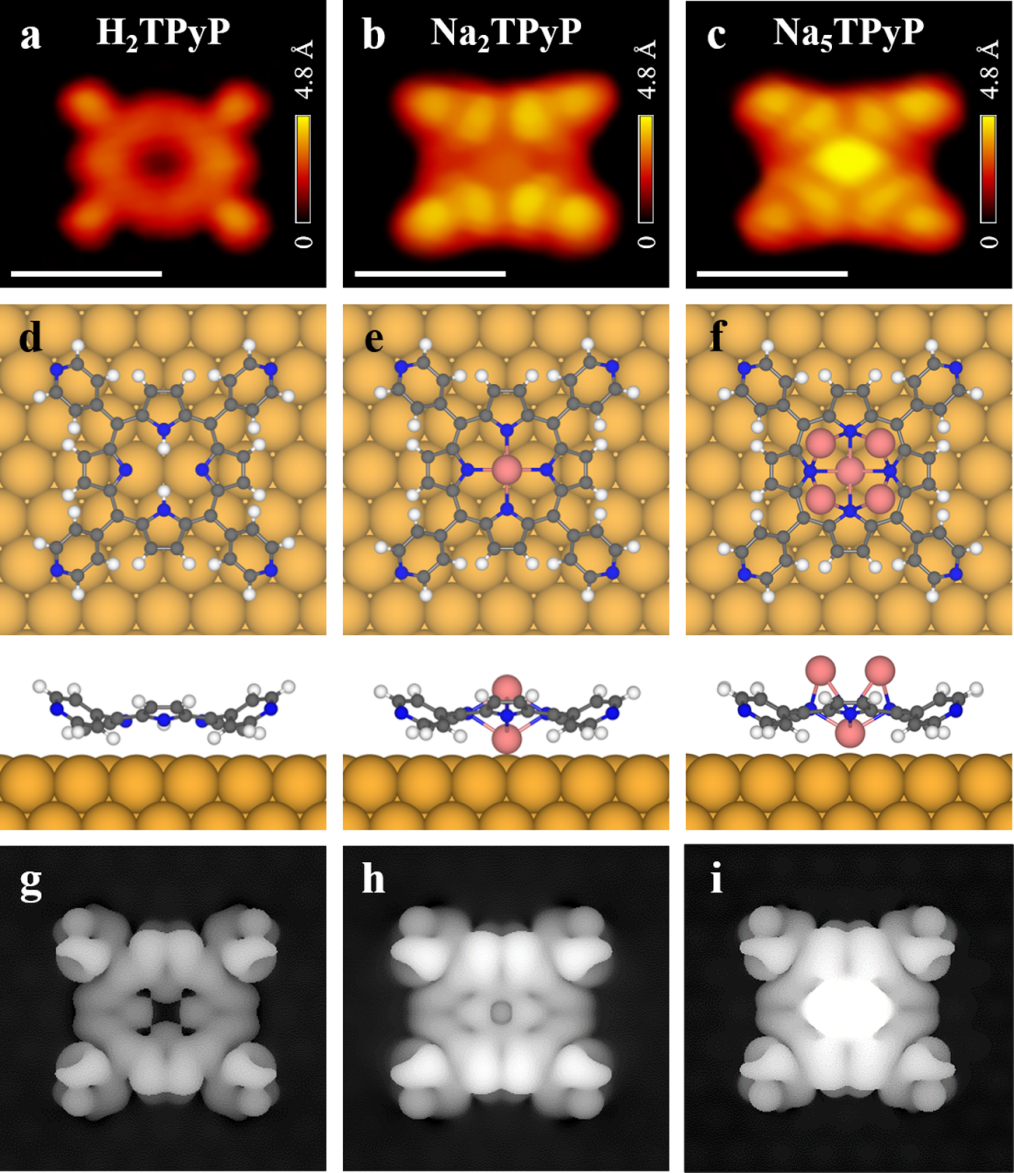
High-resolution STM images, structural models, and simulated STM images of three porphyrin-based molecules. (a–c) High-resolution STM images of (a) H_2_TPyP, (b) Na_2_TPyP, and (c) Na_5_TPyP. Scale bar: 1 nm. Scanning conditions: *V*_*t*_ = −1.0 to −1.5 V, *I*_*t*_ = 0.6 nA. (d–f) Top and side views of the corresponding DFT-optimized structural models on Au(111). H: white; C: gray; N: blue; Na: pink; Au: yellow. (g–i) Simulated STM images of (g) H_2_TPyP (*V*_*t*_ = −1.5 V), (h) Na_2_TPyP (*V*_*t*_ = −1.5 V), and (i) Na_5_TPyP (*V*_*t*_ = −1.0 V).

To validate the influence of NaCl on the fabrication of Na-porphyrins, the amount of NaCl was regulated by controlling the sublimation duration, while the coverage of H_2_TPyP was constant. First, a H_2_TPyP-precovered sample was prepared (Figure 3a), similar to the case shown in Figure 1a. Deposition of insufficient NaCl (by sublimation at ∼830 K for 1 min) followed by annealing at ∼510 K led to the appearance of bright Na_5_TPyP molecules in the minority (Figure 3b). Subsequently, after deposition of additional NaCl under the same condition and annealing at ∼510 K, the proportion of bright molecules significantly increased (to ∼96.0%) and was dominant over that of intact H_2_TPyP, as shown in Figure 3c, while few dim Na_2_TPyP could be observed in both cases. Therefore, the ratio of Na_5_TPyP is highly dependent on the amount of NaCl, indicating that NaCl directly participates in the synthesis of bright molecules. In addition, as discussed above, the self-metalation did not take place under this condition, further approving the assignment of Na-porphyrins.

**Figure 3:**
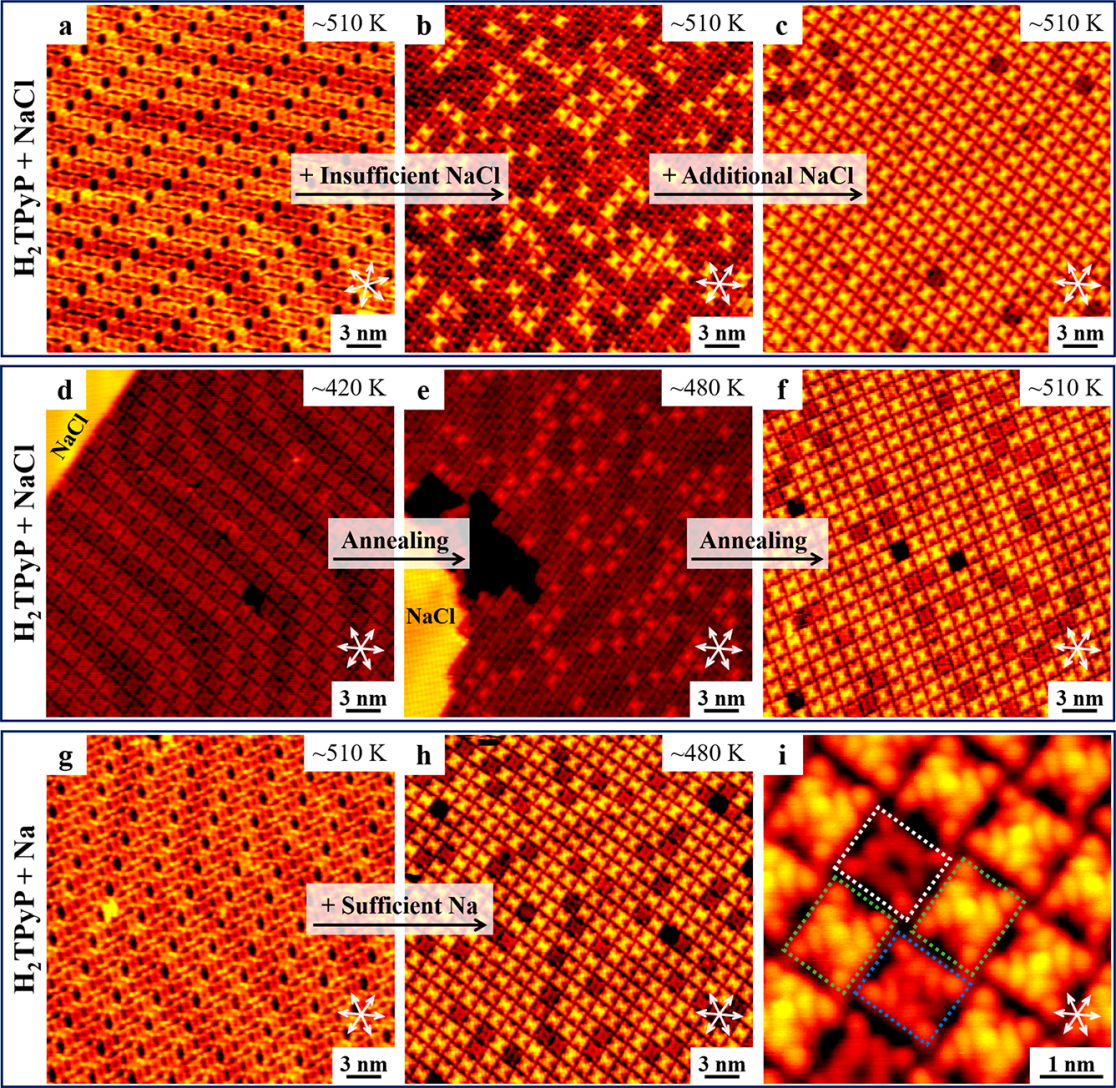
On-surface fabrication of Na-porphyrins from NaCl and Na. (a–c) NaCl-dosage-dependent metalation experiments based on H_2_TPyP and NaCl. Large-scale STM images obtained after (a) sublimation of H_2_TPyP followed by annealing at ∼510 K; (b) deposition of insufficient NaCl followed by annealing at ∼510 K; (c) deposition of additional NaCl followed by annealing at ∼510 K. (d–f) Annealing-temperature-dependent metalation experiments based on H_2_TPyP and NaCl. STM images obtained after (d) deposition of sufficient NaCl and H_2_TPyP followed by annealing at ∼420 K; (e) subsequent annealing at ∼480 K; and (f) further annealing at ∼510 K. (g–i) Metalation experiments based on H_2_TPyP and Na. (g) STM images obtained after sublimation of H_2_TPyP followed by annealing at ∼510 K. (h) Large-scale and (i) close-up STM images showing the formation of metalated molecules after the deposition of sufficient Na followed by annealing at ∼480 K. Scanning conditions: *V*_*t*_ = −1.2 to −1.5 V, *I*_*t*_ = 0.6–0.8 nA.

Thereafter, another key point is to figure out whether deprotonation of N–H groups occurred at the macrocycles of dim and bright molecules, which would lead to the formation of M-porphyrins or “sitting atop complexes” (SAT complexes),^32,33^ respectively. Annealing-temperature-dependent metalation experiments involving NaCl were thus conducted (Figure 3d–f). With sufficient NaCl, H_2_TPyP molecules remained intact after annealing the sample at ∼420 K (Figure 3d). When the sample was heated up to ∼480 K, only a few metalated ones appeared (Figure 3e), and the ratio greatly increased at ∼510 K (Figure 3f). It is worth noting that Na atoms from NaCl are capable of interacting with H_2_TPyP via ionic interactions (within Na and N) at ∼300 K, which is evidenced by the square-pattern molecular arrangement (Figure 1b and e). Moreover, after directly dosing pure metal Na to the H_2_TPyP-precovered Au(111) sample, or in a reverse order, deposition of H_2_TPyP to the Na-precovered Au(111) sample at ∼300 K, Na-based metal–organic nanostructures formed with the involvement of intact H_2_TPyP molecules (which will be discussed elsewhere), while no SAT complexes were observed. In both cases (i.e., NaCl and Na), high annealing temperatures are required to form the bright molecules (the metalation with pure metal Na will be discussed in the following text), while the formation of SAT complexes should be possible at room temperature (RT) as only electrostatic interactions are formed without intramolecular dehydrogenation. The high thermal stability of the bright species that can endure annealing temperatures of ∼600 K also supports the situation of metalation. Besides, individual dim and bright molecules diffuse as whole entities, indicating the stability of both structures. Moreover, according to previous reports,^7,10–18^ integration of metal centers into porphyrins on surfaces is always accompanied by intramolecular dehydrogenation within macrocycles. Therefore, the newly formed metalated structures were attributed to the dehydrogenated Na-porphyrins instead of SAT complexes.

Furthermore, to exclude the possibility of involvement of Cl in the synthesis of metalated molecules, pure Na was applied to verify its feasibility in the metalation of porphyrins. After the deposition of sufficient Na onto the H_2_TPyP-precovered sample (Figure 3g) followed by annealing at ∼480 K, a majority of the molecules turned bright (Figure 3h), identical to the situation shown in Figure 3f. The magnified STM image (Figure 3i) revealed that both Na-porphyrins obtained in this case shared the same characteristics as that displayed in the case of NaCl (Figure 1f). Therefore, such a control experiment reveals that the newly formed dim and bright molecules are the products of metalation with Na embedded, while Cl is not involved. Accordingly, the composition of adsorption of Cl on the top of NaTPyP, which is often the case in previous reports such as FeTPPCl^34^ and ClAlPc,^35^ can be ruled out herein. Moreover, in both cases (i.e., NaCl and Na), two hierarchical interaction steps have been observed, that is, Na first gathers H_2_TPyP molecules via intermolecular ionic interactions at ∼300 K, and then residual Na enters the center of macrocycles to accomplish the metalation process at much higher temperatures. A high yield of bright Na_5_TPyP was obtained in all of the situations shown in Figure 3c,f,h, which ranges from ∼87.2% to ∼96.0%. It is also noteworthy that in both cases the dim Na_2_TPyP accounts for a small ratio (in the range of 1.1%–2.5%), indicating less stability compared to that of bright Na_5_TPyP.

To experimentally explore the structure of bright molecules, STM lateral manipulations^36,37^ were conducted on the bright ones (as depicted by green solid rectangles in Figure 4) in a controllable line-scan mode, typically by lowering the tip bias voltage to ∼−15 mV and increasing the tunneling current to ∼5 nA. As shown in Figure 4a–c, the bright molecules A and B were manipulated sequentially, and both were controllably transformed to the dim ones (in blue dotted rectangles, labeled as A’ and B’) via removal of additional Na atoms. As the next step, the bright protrusions were intentionally moved from the bright molecules to the dim ones by STM manipulations, along the directions of the white arrows in Figure 4d and e (that is, from molecules C to D and E to C’, respectively). Interestingly, the bright molecule C was first converted to the dim C’ and then back to the bright C” (with the same morphology compared to the initial one), accompanied by the conversions from the dim D to bright D’ and from the bright E to dim E’ (Figure 4d– f). Consequently, the interconversion between bright Na_5_TPyP and dim Na_2_TPyP structures was repeatedly achieved, and the additional Na atoms were successfully transferred within the three highlighted molecules (i.e., C, D, and E). Notably, controlled adsorption of peripheral substituents (for instance, halogen atoms^34^ and N atoms^38^) on the metal center or desorption from it may lead to some intriguing physical or chemical phenomena, such as Yu–Shiba–Rusinov resonances and changes in oxidation states. Hence, STM manipulations on central top-layer Na atoms herein present promising prospects in precisely modifying the structures, which would tune the corresponding properties of porphyrins.

**Figure 4:**
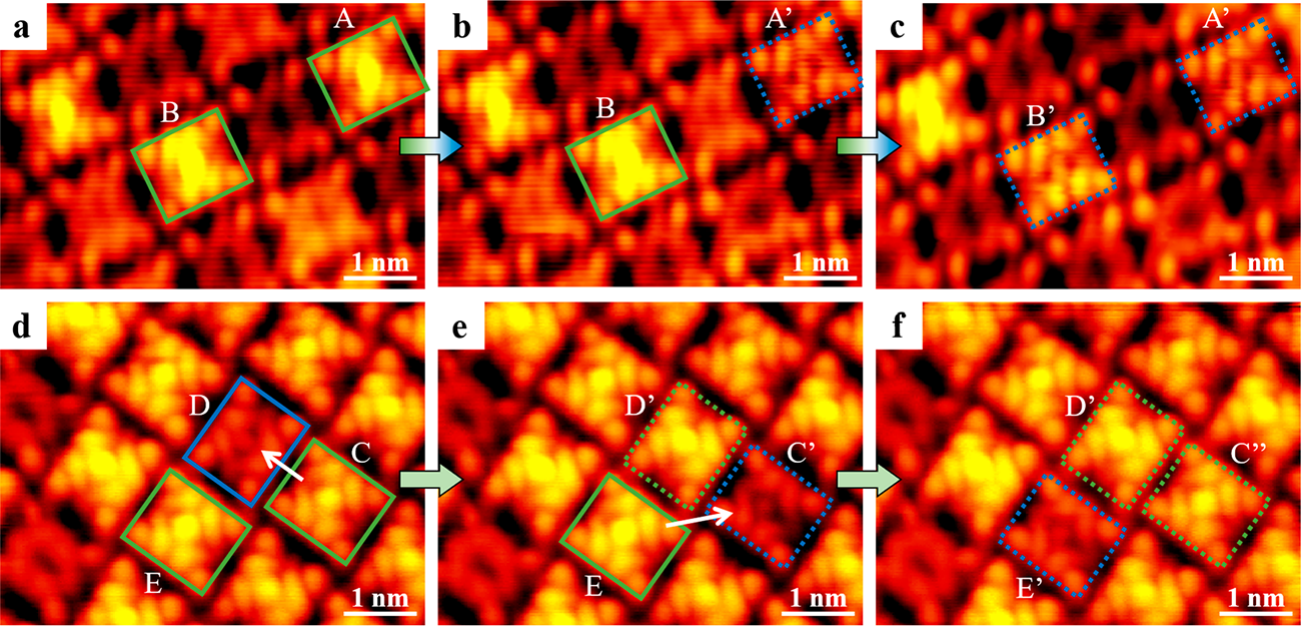
STM lateral manipulations on Na_5_TPyP. STM images recorded at the same regions showing (a–c) the transformation from Na_5_TPyP to Na_2_TPyP and (d–f) the interconversion between Na_5_TPyP and Na_2_TPyP by STM lateral manipulations, where Na_5_TPyP and Na_2_TPyP molecules are depicted by green and blue rectangles, respectively. The molecules before and after manipulations are highlighted in solid and dotted rectangles, respectively. Scanning conditions: *V*_*t*_ = −1.2 V, *I*_*t*_ = 0.6 nA.

In summary, by the combination of STM imaging/manipulations and DFT calculations, we presented the successful fabrication of two Na-porphyrins, Na_2_TPyP and Na_5_TPyP, on Au(111) by introducing inorganic salt NaCl as an easily available Na supplier. The metalation scenario was further verified by dosing pure Na experimentally as well as theoretical calculations. Moreover, by STM manipulations, the interconversion between Na_5_TPyP and Na_2_TPyP could be controllably achieved. These findings elucidate the feasibility of metalation with inorganic salt, which would serve as a promising strategy to embed various metal components into porphyrin-based structures with further functionalization and modification for potential applications in molecular magnetism, heterogeneous catalysis, etc.

## Supplementary Material



## Notes

Experimental methods, additional STM images, and DFT calculations (PDF)
