# Supplementary material for: On-Surface Synthesis of Na-Porphyrins Using NaCl as a Convenient Na Source
Source: Precis Chem. 2023 Apr 5;1(4):226–32. doi: 10.1021/prechem.3c00014 (PMC12382233; doi:10.1021/prechem.3c00014)
Supplement: Supplementary file 1 [file pc3c00014_si_001.pdf]

Supporting Information

On-Surface Synthesis of Na-Porphyrins Using NaCl as a Convenient Na Source

Zewei Yi, Chi Zhang*, Zhaoyu Zhang, Rujia Hou, Yuan Guo, Wei Xu*

Interdisciplinary Materials Research Center, School of Materials Science and Engineering, Tongji University, Shanghai 201804, People's Republic of China.
*E-mail: xuwei@tongji.edu.cn, zhangchi11@tongji.edu.cn

Table of contents:

Methods

Supplementary STM images and DFT calculations

References

Methods

All STM experiments were performed in a UHV chamber (base pressure ~1.0×10^{-10} mbar) equipped with a variable-temperature, fast-scanning "Aarhus-type" STM using electrochemically etched W tips purchased from SPECS.[1,2] The Au(111) substrate was cleaned by Ar^+-ion sputtering and annealing at ~800 K for repeated cycles. After thorough degassing, H_2TPyP (purchased from Macklin, purity > 97%) and NaCl (purchased from Bidepharm, purity > 99.9%) were evaporated separately using home-made Knudsen-cells at ~650 K and ~830 K, respectively. The alkali metal sodium was evaporated from Alvasource (from Alvatec) via conventional resistance heating after fully degassing. For the annealing procedures in sample preparation, the sample was kept at the target temperature for 10 minutes. After preparation, the sample was transferred within the UHV chamber to the STM head, where measurements were carried out at room temperature. All the STM images were further smoothed to eliminate noises.

The calculations were performed in the framework of DFT by using the Vienna ab initio simulation package (VASP).[3,4] The projector-augmented wave method was used to describe the interaction between ions and electrons.[5,6] The Perdew−Burke−Ernzerhof generalized gradient approximation exchange-correlation functional was employed,[7] and van der Waals interactions were included using the dispersion-corrected DFT-D3 method of Grimme.[8] The atomic structures were relaxed using the conjugate gradient algorithm scheme as implemented in the VASP code until the forces on all unconstrained atoms were ≤ 0.03 eV/Å. Plane waves were used as a basis set with an energy cutoff of 400 eV. Au(111) substrate was modelled by three-layered slabs separated by a ~15 Å vacuum region for the structural models, where the bottom one layer was fixed. Simulated STM images were obtained based on the Tersoff−Hamann method.[9,10]

Supplementary STM images and DFT calculations

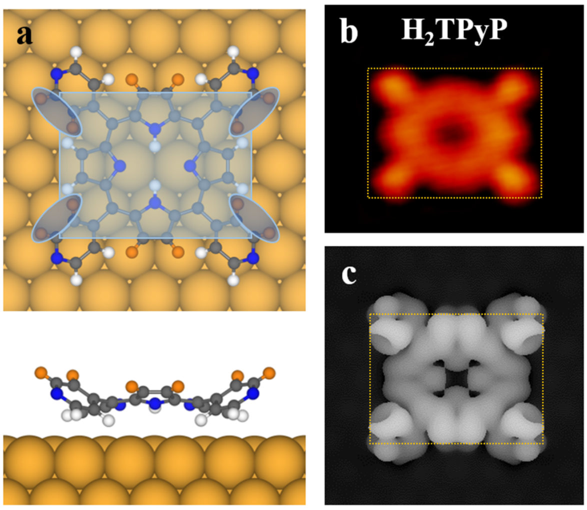

Figure S1. Comparison between the experimental and simulated STM images. (a) Top and side views of the DFT-optimized structure of H_2TPyP. C: grey; N: blue; Au: yellow. Hydrogen atoms located above and below the molecular plane are colored orange and white, respectively. The STM

morphology with prominent apparent height is shaded blue. (b) High-resolution STM image (V_t = -1.5 V, I_t = 0.6 nA) and (c) simulated STM image (V_t = -1.5 V) of H_2TPyP. The prominent molecular peripheries in the experimental and simulated images are indicated by the yellow rectangles to guide the eye.

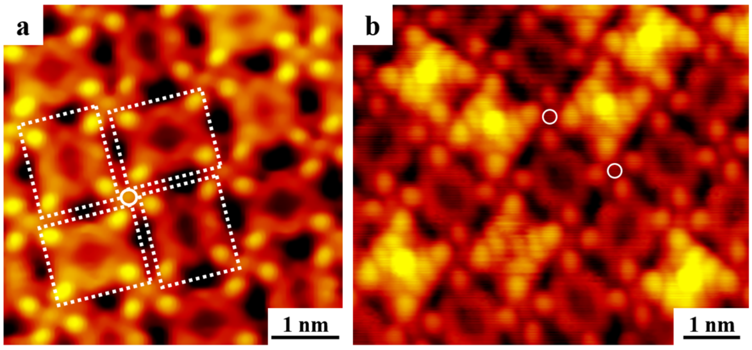

Figure S2. STM images of close-packed structures obtained with a special tip state, which are composed of (a) H_2TPyP molecules, (b) H_2TPyP molecules coexisting with some Na-TPyP molecules. The Na atoms located at the centers of four neighboring molecules were visible (as typically depicted by circles). Scanning conditions: V_t = -1.2 V, I_t = 0.6 ~ 0.7 nA.

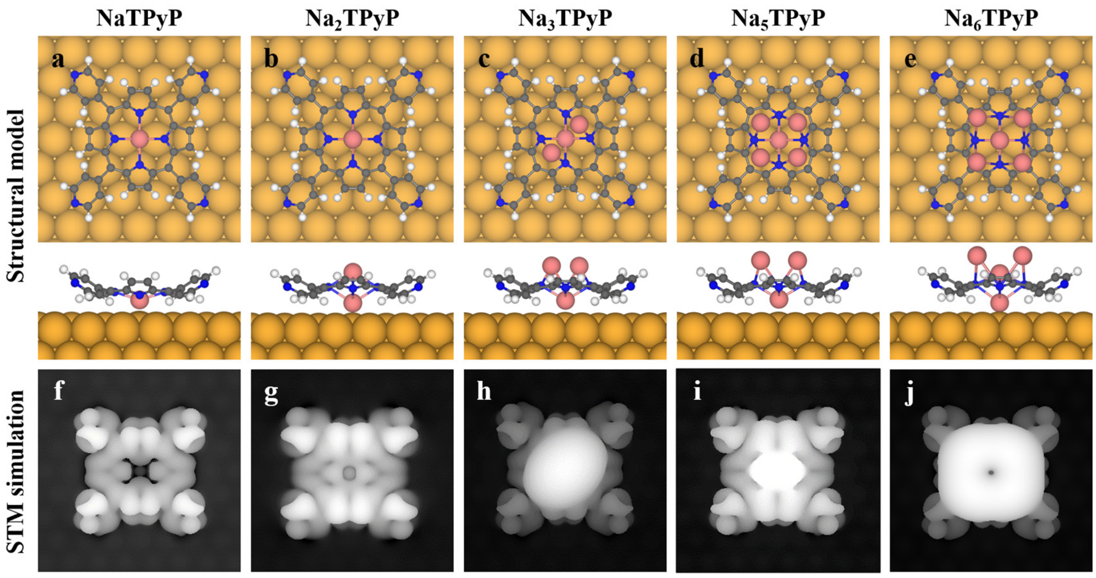

Figure S3. (a)-(e) Top and side views of DFT-optimized structural models of (a) NaTPyP; (b) Na_2TPyP; (c) Na_3TPyP; (d) Na_5TPyP; (e) Na_6TPyP on Au(111). H: white; C: grey; N: blue; Na: pink; Au: yellow. (f)-(j) Simulated STM images of (f) NaTPyP (V_t = -1.5 V), (g) Na_2TPyP (V_t = -1.5 V), (h) Na_3TPyP (V_t = -1.0 V), (i) Na_5TPyP (V_t = -1.0 V), and (j) Na_6TPyP (V_t = -1.0 V).

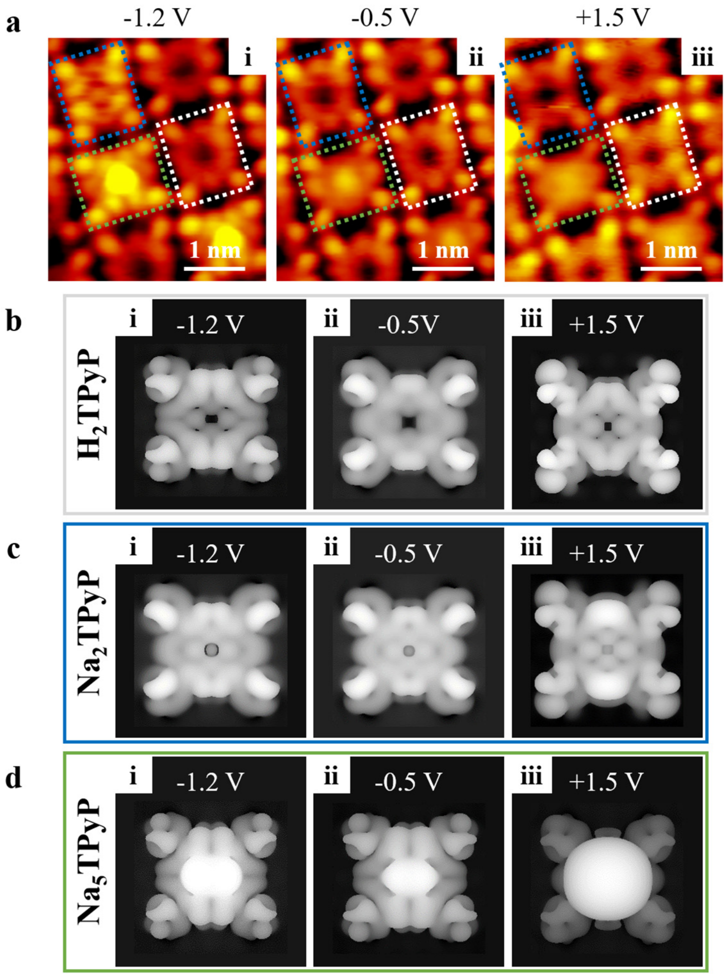

Figure S4. Comparison between STM images and DFT simulations at different bias voltages. (a) STM images showing the coexistence of intact H_2TPyP, dim Na_2TPyP, and bright Na_5TPyP molecules (depicted by white, blue, and green rectangles, respectively) recorded at the same region with different bias voltages. (b-d) Simulated STM images of (b) H_2TPyP, (c) Na_2TPyP, and (d) Na_5TPyP at different bias voltages as indicated on the images.

References

(1) Besenbacher, F. Scanning tunnelling microscopy studies of metal surfaces. *Rep. Prog. Phys.* **1996**, 59, 1737-1802.

(2) Laegsgaard, E.; Österlund, L.; Thostrup, P.; B. Rasmussen, P.; Stensgaard, I.; Besenbacher, F. A high-pressure scanning tunneling microscope. *Rev. Sci. Instrum.* **2001**, 72, 3537-3542.

(3) Kresse, G.; Hafner, J. Ab initio molecular dynamics for open-shell transition metals. *Phys. Rev. B* **1993**, 48, 13115-13118.

(4) Kresse, G.; Furthmüller, J. Efficient iterative schemes for ab initio total-energy calculations using a plane-wave basis set. *Phys. Rev. B* **1996**, 54, 11169-11186.

(5) Blöchl, P. E. Projector augmented-wave method. *Phys. Rev. B* **1994**, 50, 17953-17979.

(6) Kresse, G.; Joubert, D. From ultrasoft pseudopotentials to the projector augmented-wave method. *Phys. Rev. B* **1999**, 59, 1758-1775.

(7) Perdew, J. P.; Burke, K.; Ernzerhof, M. Generalized Gradient Approximation Made Simple. *Phys. Rev. Lett.* **1996**, 77, 3865-3868.

(8) Grimme, S.; Antony, J.; Ehrlich, S.; Krieg, H. A consistent and accurate ab initio parametrization of density functional dispersion correction (DFT-D) for the 94 elements H-Pu. *J. Chem. Phys.* **2010**, 132, 154104.

(9) Vanpoucke, D. E. P.; Brocks, G. Formation of Pt-induced Ge atomic nanowires on Pt/Ge(001): A density functional theory study. *Phys. Rev. B* **2008**, 77, 241308.

(10) Tersoff, J.; Hamann, D. R. Theory of the scanning tunneling microscope. *Phys. Rev. B* **1985**, 31, 805-813.
